# Syphilitic Affections of the Nervous System

**Published:** 1874-11

**Authors:** Edward N. Brush


ART. III.—*Syphilitic Affections of the Nervous System.* By EDWARD N. BRUSH, M. D.

(Continued from Page 92.)

CASE I.—S——, aged 29, contracted syphilis in 1868. This was followed by syphilitic eruptions, loss of flesh, alopecia, sore throat, rheumatic pains, etc. From these he gradually improved under the use of mercury, and later, in the history of the case, iodide of potassium. In Dec. 1872, he had hemiplegia without loss of consciousness, together with slight mental disturbance. The hemiplegia was preceded by premonitory signs, to which he, however, gave no attention. He first noticed a slight feeling of pain in the head, followed by more severe pain, and vertigo. His memory began to fail slightly, so that he could not apply himself to business, with his usual vigor. Impairment of motion was hardly perceptible at first, but gradually came on. He paid no attention to this, thinking it rheumatic in character. Upon attempting to rise from his chair, one morning, he found that his control over one side was entirely gone. There was slight ptosis, the mouth was drawn to one side, and sensation was also diminished. The muscles of the tongue were so much affected, that speech was considerably impaired. He was put upon the use of iodide of potassium, in twenty grain doses, three times daily, with proto-iodide of mercury, one grain at night. Under this treatment he rapidly improved up to a certain point, so that he was able, throughout the following summer, to resume his ordinary occupation as a book-keeper. Since this time his improvement has been more gradual. There still remains upon the affected side slight impairment of motion and sensation. The mouth and face has resumed its natural shape, and he speaks without difficulty, there being no perceptible defect in his voice.

Case II.—E. O. ———, married, aged 41, contracted syphilis eighteen years ago. The secondary symptoms manifested themselves in a short time, for which he underwent treatment with benefit. In June, 1873, he applied for treatment for extensive ulceration of the throat, accompanied by severe nocturnal pains in his limbs, and headache. He was placed upon iodide of potassium, ten grains, three times daily, and given some mild local application for his throat. The patient was seen from time to time, until November, at which time the throat difficulty had entirely subsided, resulting, however, in a loss of a small portion of the soft palate, and consequent impairment of the voice. The severity of his attacks of headache had somewhat diminished, but he was not altogether free from them. In the early part of March, 1873, while sitting in a chair at home, he experienced a sensation, which he discribed as a shock of electricity, upon his left side. He found, upon attempting to move, that he had lost all control over that side. He fell from his chair, but at no time lost consciousness. He summoned a physician, who treated him upon the supposition, that it was apoplectic in character. I have been unable to ascertain what remedies were used, but at no time was he treated with the supposition, that the lesion might be of syphilitic character. He improved to a certain extent, so much so, that he was able to walk with a crutch and cane for a short distance. In Sept., 1873, becoming weary of the slow improvement, he concluded to change his physician, and accordingly came to Dr. Miner's office. Having previously treated him for syphilis, it immediately occurred to Dr. M., that the paralysis might be of syphilitic origin. Mr. O., at this time walked with a crutch and cane, dragging his left foot and leg heavily. He was subject to severe and frequently repeated attacks of headache, frequently being kept awake nearly twenty-four hours by them. So long a time having elapsed since the hemiplegic attack, a guarded prognosis was given, but there was not much hesitation in promising some improvement.

Iodide of potassium in twenty grain doses was prescribed three times daily, this amount to be increased as the patient would tolerate it. In a short time improvement was manifested, both, in the general appearance of the patient and, in the condition of the side. At this date he considers himself well as far as the paralysis is concerned, but a careful examination reveals a slight loss of power, which will probably be permanent. He is also subject to occasional turns of nervousness, at which times he is unable to attend to his business for a day or two; these are however, becoming less frequent, and will probably wholly disappear.

The iodide of potassium was increased up to forty grains, three times daily, and was then gradually diminished, until he is now taking ten grains, with occasional intermissions.

This is a case of considerable interest, as showing the value of proper treatment in this class of cases. His first attendant was aware of his having had syphilis several years ago, and while he thought of it as a probable cause of the paralysis, did not modify his treatment to accord with that idea.

In all cases of a doubtful character, as this was, it certainly can do no harm, and may be of incalculable benefit, to give the patient the benefit of the doubt. As to the character of the lesion present, nothing definitely can be stated, as the patient was not seen until some months after the attack; but from the headache, which was so severe and persistent, I am inclined to think the paralysis due to the presence of a gummy tumor.

Case III.—J——, aged 50, contracted sypnilis some seven years since. This was followed by iritis, eruption, loss of hair, etc. In January and Feb. 1873, he noticed nodes upon his clavicle and tibia, which disappeared on the free administration of iodide of potassium. On the morning of March 10, 1873, having to walk a short distance through the snow, he found some difficulty in the left leg, being unable to move it through the snow as well as the right one. The impairment of motion was however but slight, the most noticeable and distressing feature being a complete numbness of the entire left side. This numbness did not come on suddenly, but had been noticed slightly on the day previous, and did not deter the patient from pursuing his business until late in the afternoon, when he found it necessary to take to his bed. In a short time the condition of anæsthesia was superceded by one of hyperæsthesia. Under constitutional treatment a rapid improvement was made, the patient gained in flesh and general health, but the perverted sensation has never been entirely corrected, the upper portion of the body has become nearly normal in sensibility, but the lower extremity still remains considerably impaired.

There are some points of interest in this case but time will only permit mention of but few. The first is the manner of inoculation of syphilis, the lesion being upon one of the fingers from a man the subject of secondary syphilis.

The question also arises, what was the particular nervous lesion in this case. It seems to have been one of the cases denominated nervous symptoms *sine materia.* During the whole course of the disease there were no symptoms which would point to any particular lesion. No headache, dullness of intellect, nor any brain symptoms in the least. The term *sine materia* does not express exactly the condition of affairs probably as they often exist during the course of the disease, yet on *post mortem* no material lesion of the brain can be found. Keyes is inclined to the opinion that in these cases there is a temporary local congestion, sufficient to product the nervous symptoms and if not controlled to leave permanent impairment of function, but not of sufficient force to produce any changes appreciable on *post mortem* examination. In an earlier portion of this paper the idea has been expressed that reflex irritation might be capable of producing these symptoms. It is well known that cases of epilepsy are produced by the irritation of an indolent sore or old cicatrix, and that coma, convulsions, and paralysis are caused by gastric or intestinal irritation, and it seems highly probable that a syphilitic nodule situated in the liver or in any of the other thoracic or abdominal organs, might be productive of as serious and varied nervous symptoms.

CASE IV.—John J——, colored, whitewasher, aged 44 had chancre at 30, and afterwards general syphilis.

When first seen in April, 1873, he gave no history of syphilitic disease. His condition was as follows: He complains of want of power and sensation in his lower extremities; says they feel "as if asleep." He can walk without the aid of a cane but his gait is shuffling and uncertain. He has been treated by two or three physicians for his trouble, which has been gradually growing upon him for about three months. The treatment which he has received and his account of what was said by his medical attendants, seems to indicate that they viewed the case as one of progressive spinal sclerosis. It was some weeks before the patient could be induced to remember any former syphilitic taint, and much ingenuity had to be exercised to gain his former history. He was put upon the iodide of potassium, and also ordered to take three grains of blue pill at night for a short time. He did not improve and some doubt existed as to the correctness of the diagnosis. In a short time he became almost wholly paraplegic so that he was confined to his bed; his bladder also was affected so that his urine had either to be drawn, or flowed away without the patients knowledge.

With slight additions in the shape of sedatives and tonics the treatment was continued as before, the iodide of potassium being increased to about two drachms daily in divided doses. After being confined to his room a few weeks a considerable improvement was noticed, which continued until he was able in a little less than two months to resume his business, and he may now be seen every day on the streets walking and drawing his whitewash cart after him.

These four cases illustrate admirably the general character of syphilitic affections of the nervous system. Others might be reported, but they would only be repetitions of the same facts. Success in a great measure has attended the treatment in each case, but others might be reported where the issue was not so desirable; it may, however, be safely stated that the treatment of nervous affections due to syphilis is attended by as good or even better results than in any other instance. One object of this report was to call attention to the fact that these cases are more common than many suppose, and to urge the importance of obtaining fully the patients former history. His word can never be taken as evidence, unless a full account of his former ailments is obtained; it should not be forgotten also, that inherited syphilis gives rise to nervous affections and therefore, if possible, the history of the parents should be obtained. The press of other duties has made this paper deficient in many particulars, it is hoped, however, that it will not prove altogether without value.

